# Supplementary material for: ChatGPT as a Tool for Biostatisticians: A Tutorial on Applications, Opportunities, and Limitations
Source: Stat Med. 2025 Oct 23;44(23-24):e70263. doi: 10.1002/sim.70263 (PMC12548020; doi:10.1002/sim.70263)
Supplement: Supplementary file 1 — Data S1. Supporting Information. [file SIM-44-0-s001.zip › Supplementary Materials Additional.zip!/3.1_Systematic_Reviews_and_Meta_aspects/3.1_Forest Plot - 4o - Chat 2.pdf]

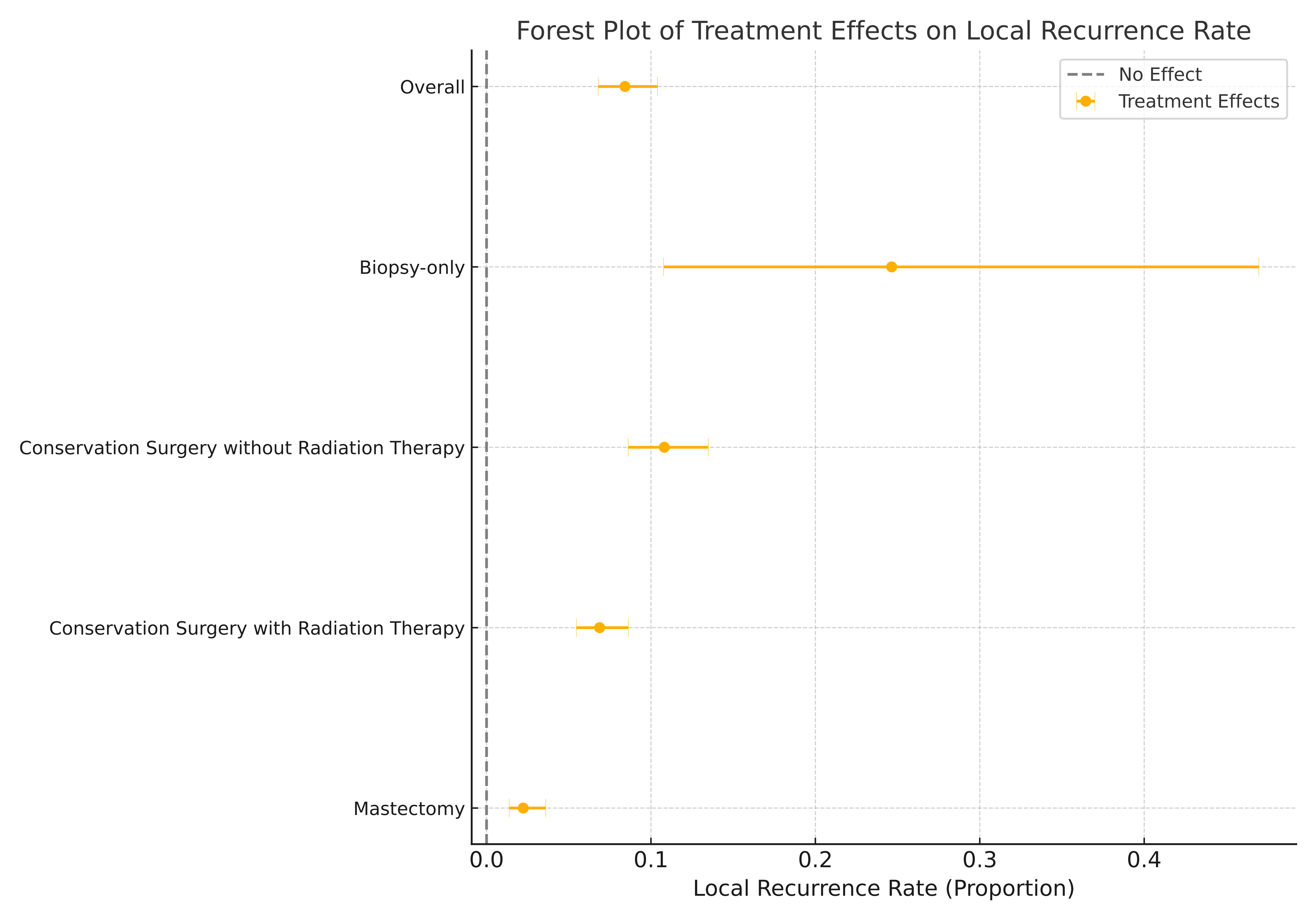
Forest Plot of Treatment Effects on Local Recurrence Rate
No Effect
Treatment Effects
Overall
Biopsy-only
Conservation Surgery without Radiation Therapy
Conservation Surgery with Radiation Therapy
Mastectomy
0.0
0.1
0.2
0.3
0.4
Local Recurrence Rate (Proportion)
